# Several grain dietary patterns are associated with better diet quality and improved shortfall nutrient intakes in US children and adolescents: a study focusing on the 2015–2020 Dietary Guidelines for Americans

**DOI:** 10.1186/s12937-017-0230-0

**Published:** 2017-02-20

**Authors:** Yanni Papanikolaou, Julie Miller Jones, Victor L. Fulgoni

**Affiliations:** 1 Nutritional Strategies Inc., 59 Marriott Place, Paris, ON N3L 0A3 Canada; 2 Distinguished Scholar and Professor Emerita of Food and Nutrition, Department of Foods and Nutrition, St. Catherine University, 4030 Valentine Ct. Arden Hills, Minnesota, MN 55112 USA; 3 Nutrition Impact, LLC, 9725 D Drive North, Battle Creek, MI USA

**Keywords:** NHANES, Grains, Children, Adolescents, Nutrient intakes, Diet quality

## Abstract

**Background:**

The present study identified the most commonly consumed grain food patterns in US children and adolescents (2–18 years-old; *N* = 8,367) relative to those not consuming grains and compared diet quality and nutrient intakes, with focus on 2015–2020 Dietary Guidelines for Americans (2015–2020 DGA) shortfall nutrients.

**Methods:**

Cluster analysis using data from the National Health and Nutrition Examination Survey 2005–2010, identified 8 unique grain food patterns: a) no consumption of main grain groups, b) cakes, cookies and pies, c) yeast bread and rolls, d) cereals, e) pasta, cooked cereals and rice, f) crackers and salty snacks, g) pancakes, waffles and French toast and other grains, and h) quick breads.

**Results:**

Energy intake was higher for all grain cluster patterns examined, except ‘cereals’, compared to no grains. Children and adolescents in the ‘yeast bread and rolls’, ‘cereals’, ‘pasta, cooked cereals and rice’, and ‘crackers and salty snacks’ patterns had a higher diet quality relative to no grains (all *p* < 0.01). Energy adjusted (EA) dietary fiber intake was greater in five of the seven grain patterns, ranging from 1.8 – 2.8 g more per day (all *p* < 0.01), as compared to those consuming no grains. All grain patterns, except cakes, cookies and pies had higher EA daily folate relative to children in the no grains pattern (all *p* < 0.0001). EA total fat was lower in ‘cereals’, ‘pasta, cooked cereals and rice’, and ‘pancakes, waffles, French toast and other grains’ in comparison to the no grains food pattern (all *p* < 0.01). EA magnesium intakes were greater in children and adolescents consuming ‘yeast bread and rolls’, ‘pasta, cooked cereals and rice’, and ‘quick breads’, while EA iron was higher in all grain patterns relative to no grains (all *p* < 0.01). EA vitamin D intake was higher only in children consuming ‘cereals’ vs. no grain group (*p* < 0.0001). There were no significant differences in total or added sugar intake across all grain clusters as compared to no grains.

**Conclusions:**

Consumption of several, but not all, grain food patterns in children and adolescents were associated with improved 2015–2020 DGA shortfall nutrient intakes and diet quality as compared to those consuming no grains.

## Background

The 2015–2020 Dietary Guidelines for Americans (2015–2020 DGA) policy report states that several nutrients are under-consumed relative to requirement levels set by the Institute of Medicine (IOM). These have been characterized as shortfall nutrients and include vitamin A, vitamin D, vitamin E, vitamin C, folate, calcium, magnesium, fiber, and potassium. For adolescent, premenopausal females and women who are pregnant, iron is also deemed an under-consumed nutrient of public health concern largely due to increased risk of iron-deficiency in these populations. Of the shortfall nutrients, calcium, vitamin D, fiber, and potassium also are classified as nutrients of public health concern because their under-consumption has been linked in the scientific literature to adverse health outcomes [1]. The report further identified that a healthy dietary pattern is higher in fruits, vegetables, whole grains, low- and non-fat dairy, seafood, legumes, and nuts; and lower in red and processed meat, sugar-sweetened foods and beverages and refined grains. However, a variety of grain-based food products, of which include refined/enriched grains, are sources for several shortfall nutrients identified by the DGA, including dietary fiber, folate, iron, and magnesium [1]. With mandatory folic acid fortification commencing in 1998 by the Food and Drug Administration [2], specific grain foods became leading sources for folate; breads, rolls, and crackers are the largest contributor of total folate to the US diet, contributing nearly 16% of total intake, which exceeds contribution of folate from vegetables [3]. Similarly, using data from the National Health and Nutrition Examination Survey (NHANES) 2003–2006, researchers have reported that fortification of grain foods substantially contributes nutrient adequacy for U.S. children and adolescents aged 2–18 years-old, without excessive intakes for most vitamins and minerals [4].

While certain grain food products are contributors of nutrients to limit in the diet, including added sugar, total and saturated fat [5, 6], grain foods also contribute positive nutrients to the diet, including dietary fiber, iron, magnesium, and B vitamins (thiamin, riboflavin, niacin and folate). Food sources of energy and nutrients data in children showed that while three of the top ten ranking foods for calorie contribution to the diet were grain-based foods, the top ten ranking food sources of dietary fiber included six grain-based products, collectively contributing 40% of total daily dietary fiber intake [4]. Others have argued that while three of the top ten sources of energy provided no nutritional value, the remaining sources of energy, including milk, beef, poultry, cheese and baked goods are significant contributors of nutrients of concern and other essential nutrients, postulating the premise that elimination of these foods from food patterns could potential have inadvertent effects on diet quality in the US population [6].

Recent NHANES data in adults showed that some, but not all, grain food patterns were associated with better nutrient intakes, improved diet quality and beneficial obesity-related parameters [7]. While 2015–2020 DGA identify several healthy dietary food patterns, and encourage increased whole grain consumption and reduced refined grain intake, at present, there are no data that evaluate the association of different grain food patterns on nutrient intakes and diet quality outcomes in children and adolescents. Further, some popular diet plans encourage diet patterns that omit gluten- containing or grain-based foods (e.g. Paleolithic diet) as healthier patterns [8, 9]. As such, the objective of the current analyses was to isolate the most commonly consumed grain food patterns in U.S. children and adolescents and compare nutrient intakes and diet quality of those consuming various grain food patterns to those not consuming grain foods using data from the National Health and Nutrition Examination Survey (NHANES) 2005–2010. The hypothesis for the present analysis was that certain grain food patterns are associated with improved diet quality and can significantly contribute nutrients, including shortfall nutrients, while concurrently lowering nutrients to limit in the diet.

## Methods

Data were obtained from What We Eat in America, the dietary intake component of NHANES. NHANES is a government-directed program led by the Center for Disease Control and Prevention in collaboration with US Department of Agriculture. Written informed consent was obtained for all participants or proxies, and the survey protocol was approved by the Research Ethics Review Board at the National Center for Health Statistics. **The distribution of the civilian** **non-institutionalized** **US population, as well as response rate percentages and population totals in NHANES 2005–2010 data by age and gender, can be viewed at** **www.cdc.gov/nchs/nhanes/response_rates_cps.htm.** Data from the current NHANES are released every two years and for the current analyses, we used three data releases, namely 2005–2006, 2007–2008, and 2009–2010 [10, 11].

**The dietary intake data were obtained from an** **in-person** **24-hour** **dietary recall (Day 1) by trained specialists using the Automated** **Multiple-Pass** **Method** **[** 12 **]** **as a means to reduce bias in reporting energy and nutrient** **intakes in the Mobile Examination Center. The** **Multiple-Pass** **Method consisted of five steps: (1) the quick list, which included an uninterrupted list of foods and beverages consumed by the subject; (2) the forgotten foods list, which queried the subject on categories of foods that have been documented as frequently forgotten; (3) a time and occasion where foods were consumed; (4) the detail cycle, which elicited descriptions of foods and amounts consumed with the aid of an interactive Food Model Booklet and measuring guides; and (5) final probe review.** USDA’s Food and Nutrient Database for Dietary Studies, 3.0, 4.1, and 5.0 was used to code dietary intake data and calculate nutrient intakes [13–15].

Cluster analysis was used to develop patterns of grain consumption—a statistical procedure that analyses large data sets to identify various patterns while trying to maximize differences among the patterns. **Cluster analysis allows for the focus on a specifically defined aspect (i.e., grain food consumption) and then forces maximal differences in clusters for assessments. Cluster analysis also allows for group comparisons rather than factor analysis which are generally associations.** The USDA food coding system was used to define categories of grain foods [15]. Grain foods intake patterns were identified using SAS 9.2 (SAS Institute, Cary, NC, 2013) PROC CLUSTER using a single 24-hour dietary recall in NHANES 2005–2010. **SUDAAN v.11.0 (Research Triangle Institute; Raleigh, NC) was used to adjust analyses for sampling weights and the sampling units and strata information as provided by NHANES.** Clusters were developed based on the percentage of calories consumed from the grain products as the centroid for each cluster. Grains from flour and dry mixes, mixed dishes, and meat substitutes were not included in development of grain clusters. Cluster analyses provides the ability to focus on a particular defined aspect (e.g. calories from grains) and then forces maximal differences in clusters for assessment. For these analyses, the USDA grains products main categories were used to identify the grain cluster patterns of intake (see Table 1).

**Table 1:** Grain cluster pattern based on percentage of calories from grains in children and adolescents 2–18 years-old of age using data from NHANES 2005–2010

Cluster Number	Grain Foods Pattern	Description
0	No Grains	4.0% of the population
1	Cakes, Cookies and Pies	(5.1% of the population) with approximately 92% of grains coming from this grain group)
2	Yeast Breads and Rolls	(33.8% of the population) with over 68% of grains coming from this grain group;
3	Cereals	(4.0% of the population) with over 95% of grains coming from this grain group;
4	Pasta, Cooked Cereals and Rice	(4.9% of the population) with over 67% of grains coming from this grain group;
5	Crackers and Salty Snacks	(26.1% of the population) with over 53% of grains coming from this grain group;
6	Pancakes, Waffles, French Toast and Other Grains	(9.4% of the population) with over 51% of grains coming from this grain group and approximately 23% of grains coming from yeast bread and rolls.
7	Quick Breads	(12.8% of the population) with approximately 57% of grains coming from this group.

All main grain food codes fit into one and only one of the grain foods groupings. The patterns identified by the cluster analysis were then identified by percent calories within each grain food grouping (only groups that contributed 5% or more of calories were used to define the clusters) at the centroid of each cluster. Using this method resulted in seven readily identifiable grain food patterns and a no consumption of main grain groups (i.e., no grains group); creating eight unique patterns of **grain food** consumption. With grain food cluster patterns identified, and using the output from the cluster procedure, each subject was then placed in the cluster that matched most closely to the pattern of calories across the food categories.

Adjusted **least-square** **means ± SE** values for subjects were determined in each cluster using PROC REGRESS and LOGREGRESS in SUDAAN 11.0 **for dietary intakes and diet quality [Healthy Eating Index (HEI)-2010]** with various sets of covariates. Covariates for analyses of energy intake, HEI-2010 and HEI sub-components [16] were age, gender, and ethnicity. **The poverty income ratio (PIR) grouped into three categories (<1.25, 1.25‒3.49, and >3.49) and physical activity (sedentary, moderate or vigorous based on questionnaire responses), current smoking status, alcohol intake (g/d), and energy intake for** **nutrient-related** **variables (with the exception of energy intake itself or** **HEI-2010**) **also served as covariates. The PIR values reflected the federally established poverty criteria, thus a PIR of <1.25 equated to below 125% of poverty, while higher values represented the subject was from a higher income status.** The HEI-2010 provides a measure of diet quality and measures conformance to federal dietary guidance and has been predominantly used to monitor dietary practices of the US population and the low-income subpopulation. Nutrient intakes were also adjusted for energy intakes. The main comparison of interest was to compare results between the no consumption of main grain groups (cluster 0) and all other clusters. A conservative *P*-value of *p* < 0.01 was set for significance.

## Results

Eight grain clusters were identified, one of which included isolating a group of children and adolescents that did not consume any of the identified grains (4.0% of the population). The eight clusters are defined as outlined in Table 1, namely: 1) no consumption of main grain groups, 2) cakes, cookies and pies 3) yeast breads and rolls, 4) cereals, 5) pasta, cooked cereal and rice, 6) crackers and salty snacks, 7) pancakes, waffles, French toast, and 8) quick breads.

### Energy and nutrient intakes

Energy intake was significantly higher for children and adolescents in all grain pattern clusters, with the exception of ‘cereals’ (*p* = 0.089), when compared to the no grains group. The higher energy intake ranged from 416 ‒ 524 kcal/d with ‘pancakes, waffles, French toast and other grains’ and ‘quick breads’ clusters representing the greatest increase in kcal/day (Table 2).

**Table 2:** Adjusted mean (SE) nutrient and energy intake for all grain clusters using NHANES 2005–2010, 2–18 years of age

	No Grains		Cakes, Cookies & Pies			Yeast Breads and Rolls			Cereals			Pasta, Cooked Cereals & Rice			Crackers & Salty Snacks			Pancakes, Waffles, French Toast and Other Grains			Quick Breads		
Energy or Nutrient	Cluster 0		Cluster 1			Cluster 2			Cluster 3			Cluster 4			Cluster 5			Cluster 6			Cluster 7		
	LSM	SE	LSM	SE	P	LSM	SE	P	LSM	SE	P	LSM	SE	P	LSM	SE	P	LSM	SE	P	LSM	SE	P
Energy (kcal)	1497	90	1931	65	0.0009	1961	22	<0.0001	1694	65	0.0887	1913	62	0.0005	1946	29	<0.0001	2021	39	<0.0001	2021	36	<0.0001
Carbohydrate (g)	245	6.0	268	3.4	0.0016	256	1.2	0.0679	269	4.2	0.0012	272	3.4	0.0004	262	1.4	0.0046	265	2.2	0.0014	255	2.1	0.1542
Total sugars (g)	134	6.7	149	3.6	0.0594	130	1.4	0.6016	139	4.9	0.5190	130	4.4	0.6484	128	1.7	0.3738	130	2.3	0.6479	124	1.9	0.1674
Added sugars (tsp eq)	22	2.0	25	0.5	0.1791	20	0.3	0.2535	21	1.0	0.7195	19	1.1	0.1331	20	0.4	0.2152	20	0.6	0.3609	18	0.4	0.0874
Protein (g)	72	2.2	59	1.1	<0.0001	72	0.5	0.7474	70	1.5	0.2823	72	1.7	0.9931	65	0.6	0.0004	67	1.3	0.0301	68	0.9	0.0685
Total fat (g)	76	1.9	72	1.2	0.0958	71	0.4	0.0296	67	1.5	0.0004	64	1.3	<0.0001	72	0.5	0.0727	70	0.8	0.0022	74	0.8	0.4128
Total monounsaturated fatty acids (g)	27	0.8	28	0.6	0.7623	26	0.2	0.0648	24	0.7	0.0003	23	0.5	<0.0001	26	0.3	0.1789	26	0.4	0.0958	27	0.4	0.7836
Total saturated fatty acids (g)	27	0.7	25	0.5	0.0080	26	0.2	0.0313	25	0.6	0.0049	22	0.7	<0.0001	25	0.2	0.0001	24	0.5	0.0001	26	0.4	0.0311
Cholesterol (mg)	261	23.5	184	8.0	0.0018	229	4.5	0.1745	198	9.4	0.0163	211	8.1	0.0771	189	4.2	0.0074	240	9.7	0.4133	233	9.9	0.2342
Dietary fiber (g)	11	0.5	12	0.4	0.4815	13	0.2	0.0007	13	0.6	0.0052	13	0.5	<0.0001	13	0.2	0.0006	12	0.3	0.1014	14	0.3	<0.0001
Calcium (mg)	962	53.5	863	28.2	0.1005	1054	12.6	0.0898	1092	31.5	0.0388	964	31.0	0.9737	968	13.6	0.8987	1056	32.4	0.1635	1043	19.1	0.1526
Magnesium (mg)	208	8.7	204	5.0	0.6863	232	2.1	0.0089	231	4.9	0.0158	257	6.7	<0.0001	230	2.3	0.0141	209	2.9	0.8743	237	4.5	0.0009
Iron (mg)	10.6	0.3	11.8	0.3	0.0097	14.17	0.15	<0.0001	19.03	0.67	<0.0001	15.45	0.39	<0.0001	14.16	0.21	<0.0001	13.34	0.23	<0.0001	13.44	0.25	<0.0001
Zinc (mg)	9.4	0.3	8.2	0.2	0.0016	10.97	0.16	0.0001	12.74	0.41	<0.0001	11.00	0.37	0.0050	10.12	0.12	0.0322	9.23	0.22	0.7165	9.93	0.19	0.2085
Sodium (mg)	2996	87.24	2645	44.20	0.0018	3137	23.94	0.1432	3004	71.10	0.9403	3429	150.37	0.0107	3066	38.87	0.4346	3129	45.06	0.2079	3118	55.97	0.2002
Potassium (mg)	2148	91.60	2021	69.94	0.2406	2243	21.46	0.3103	2333	46.88	0.0663	2365	51.12	0.0385	2070	23.24	0.3637	2044	33.81	0.3037	2209	39.34	0.5443
Folate, DFE (μg)	361	12.60	393	13.48	0.0694	526	10.60	<0.0001	772	36.39	<0.0001	636	29.86	<0.0001	523	11.21	<0.0001	438	9.85	<0.0001	498	12.04	<0.0001
Riboflavin (Vitamin B2) (mg)	1.8	0.1	1.8	0.0	0.9836	2.1	0.03	0.0001	2.6	0.04	<0.0001	2.0	0.05	0.0171	2.0	0.03	0.0154	2.1	0.04	0.0014	2.1	0.05	0.0031
Thiamin (Vitamin B1) (mg)	1.3	0.0	1.3	0.1	0.7019	1.6	0.02	<0.0001	1.9	0.04	<0.0001	1.7	0.04	<0.0001	1.5	0.03	0.0003	1.5	0.03	0.0024	1.5	0.03	<0.0001
Total choline (mg)	266	14.4	215	5.5	0.0007	260	3.4	0.6977	245	7.7	0.2363	264	6.9	0.9240	225	2.7	0.0053	260	7.4	0.6825	256	5.8	0.5145
Vitamin A, RAE (μg)	481	34.5	536	29.2	0.1948	599	9.8	0.0016	736	21.5	<0.0001	666	39.2	0.0008	551	12.2	0.0592	650	21.7	0.0001	578	18.6	0.0192
Vitamin B12 (μg)	4.2	0.2	3.8	0.1	0.1470	5.3	0.1	0.0001	6.8	0.3	<0.0001	4.7	0.2	0.1237	4.7	0.1	0.0246	4.9	0.1	0.0255	4.8	0.2	0.0529
Vitamin B6 (mg)	1.4	0.1	1.4	0.0	0.7590	1.7	0.03	0.0004	2.2	0.1	<0.0001	1.9	0.1	<0.0001	1.6	0.03	0.0059	1.7	0.04	0.0031	1.7	0.1	0.0045
Vitamin C (mg)	70.0	5.0	78.0	6.0	0.2829	80.0	2.0	0.0690	83.9	6.1	0.0575	103.6	9.0	0.0025	80.0	2.6	0.0846	72.3	3.1	0.7126	78.7	3.0	0.1833
Vitamin D (D2 + D3) (μg)	5.0	0.5	4.7	0.3	0.5028	6.0	0.2	0.0753	7.6	0.4	<0.0001	6.0	0.2	0.1363	5.3	0.1	0.5640	5.3	0.3	0.6946	5.8	0.2	0.1939
Vitamin E as alpha-tocopherol (mg)	5.8	0.3	5.6	0.2	0.6583	5.9	0.2	0.6465	5.1	0.1	0.0198	5.9	0.4	0.7239	6.2	0.1	0.1437	5.4	0.1	0.1511	6.1	0.2	0.3859
Vitamin K (μg)	52.7	4.0	57.4	5.4	0.4934	58.2	2.6	0.3003	52.2	5.5	0.9396	76.7	9.8	0.0277	52.4	1.9	0.9567	55.9	3.3	0.5779	55.4	1.7	0.5403

NHANES 2005–2010, *N* = 8,367

*LSM*: least square mean; *SE*: standard error; *P* = *p* value of difference as compared to cluster 0 (No grains)

Covariates include age, gender, ethnicity, poverty income ratio, physical activity, current smoking status, alcohol and for all variables except Energy, the covariate of energy (kcal)

Energy adjusted nutrient intakes in the eight grain food patterns are presented in Table 2. When examining nutrients of concern, as outlined by the 2015–2010 DGA [1], no differences in calcium intake was observed in children all of the grain clusters compared to those not consuming grain foods, while dietary fiber was higher in children consuming ‘yeast breads and rolls’, ‘cereals’, ‘pasta, cooked cereals and rice’, ‘crackers and salty snacks’, and ‘quick breads’, ranging from 1.8 ‒ 2.8 g/day greater daily fiber than children and adolescents in the no grains group.

In terms of nutrients (i.e., vitamins and minerals) present naturally or added to grain foods, via either enrichment or fortification practices, nutrient intakes were higher for those in certain grain clusters. Iron intake was greater across all seven grain clusters examined, thus demonstrating the relevance of both the naturally occurring and added iron in contributing to this 2015–2020 DGA shortfall nutrient. Daily vitamin D (D2 + D3) was significantly greater only in children and adolescents consuming a ‘cereals’ grain pattern, while no significant differences were observed with potassium intakes in any of the grain clusters as compared to those not consuming grain food products. Intakes of thiamin were significantly higher for children and adolescents consuming all grain clusters, except for ‘cakes, cookies and pies’, while daily intakes of riboflavin were significantly greater all grain patterns, with the exception of ‘cakes, cookies and pies’, ‘pasta, cooked cereals and rice’ and ‘crackers and salty snacks’ when compared to those not consuming grain foods. Similarly, folate was higher (76 ‒ 411 μg/d; all *p* < 0.0001) in those in all grain food clusters, except ‘cakes, cookies and pies’, relative to the no grains cluster. Zinc intake was higher only in children and adolescents consuming ‘yeast breads and rolls’, ‘cereals’ and ‘pasta, cooked cereals and rice’ and significantly lower in those consuming ‘cakes, cookies and pies’ compared to those in the no grains cluster. Magnesium intakes were greater in children and adolescents consuming ‘yeast bread and rolls’, ‘pasta, cooked cereals and rice’, and ‘quick breads’ relative to the no grains group (Table 2).

Regarding nutrients to limit, daily saturated fat intake was significantly lower in all grain patterns examined, with the exception of ‘yeast breads and rolls’ and ‘quick breads’, compared to those not consuming grain foods, with a range of difference in saturated fat ranging from 1.5 ‒ 4.8 g less per day. Daily sodium intake was only lower (approximately 350 mg/day) in children and adolescents consuming ‘cakes, cookies and pies’ compared to the no grains cluster pattern. There were no significant differences in total and added sugar intake across all grain clusters as compared to the no grain cluster (Table 2).

### Diet quality assessment

Diet quality, as measured by USDA’s HEI-2010 is depicted in Table 3. Four of the grain clusters had significantly greater scores when compared to the no grains cluster. Specifically, those in the ‘pasta, cooked cereals and rice’ had the greatest score at 50.6 ± 1.0, while children and adolescents consuming ‘yeast breads and rolls’, ‘cereals’ and ‘crackers and salty snacks’ had scores of 46.1 ± 0.5, 48.5 ± 1.2, and 46.0 ± 0.4, respectively (all *p* < 0.001) compared to the no grains cluster (42.7 ± 0.9).

**Table 3:** Adjusted mean (SE) Total healthy eating index-2010 (HEI) and component scores for all grain clusters using NHANES 2005–2010, 2–18 years of age

	No Grains		Cakes, Cookies & Pies			Yeast Breads and Rolls			Cereals			Pasta, Cooked Cereals & Rice			Crackers & Salty Snacks			Pancakes, Waffles, French Toast and Other Grains			Quick Breads		
HEI-2010 Component	Cluster 0		Cluster 1			Cluster 2			Cluster 3			Cluster 4			Cluster 5			Cluster 6			Cluster 7		
	LSM	SE	LSM	SE	P	LSM	SE	P	LSM	SE	P	LSM	SE	P	LSM	SE	P	LSM	SE	P	LSM	SE	P
Total Vegetables	2.52	0.13	2.13	0.10	0.0327	2.16	0.05	0.0121	2.44	0.16	0.7002	2.56	0.17	0.8395	1.93	0.06	0.0001	1.74	0.10	<0.0001	2.06	0.07	0.0024
Greens and Beans	0.30	0.07	0.50	0.10	0.0784	0.62	0.05	0.0002	0.61	0.16	0.0617	1.32	0.20	<0.0001	0.54	0.04	0.0064	0.52	0.09	0.0808	0.77	0.08	0.0004
Total Fruit	1.97	0.11	2.28	0.14	0.0468	2.58	0.07	<0.0001	2.51	0.17	0.0039	2.72	0.16	0.0007	2.55	0.06	<0.0001	2.60	0.12	0.0002	2.57	0.10	0.0003
Whole Fruit	1.72	0.17	1.96	0.19	0.3720	2.31	0.08	0.0004	2.38	0.23	0.0073	2.51	0.18	0.0081	2.21	0.07	0.0038	2.33	0.14	0.0079	2.25	0.09	0.0087
Whole Grains	0.26	0.06	0.57	0.10	0.0033	2.11	0.08	<0.0001	2.24	0.17	<0.0001	2.58	0.28	<0.0001	2.36	0.12	<0.0001	1.44	0.15	<0.0001	1.66	0.12	<0.0001
Dairy	6.84	0.26	6.53	0.25	0.3773	7.20	0.09	0.2028	8.09	0.29	0.0029	6.78	0.22	0.8751	6.76	0.11	0.7361	6.89	0.21	0.8796	7.12	0.16	0.3282
Total Protein Foods	3.65	0.14	3.03	0.14	0.0002	3.74	0.05	0.4854	3.38	0.16	0.1844	3.66	0.15	0.9260	3.36	0.05	0.0363	3.59	0.09	0.7878	3.47	0.08	0.3256
Seafood and Plant Protein	0.98	0.13	1.10	0.16	0.4806	1.46	0.08	<0.0001	0.98	0.18	0.9839	1.45	0.21	0.1112	1.37	0.06	0.0161	1.14	0.09	0.2582	1.47	0.09	0.0018
Fatty Acid Ratio	3.31	0.23	3.84	0.23	0.1020	3.30	0.08	0.9556	3.06	0.34	0.5560	4.15	0.31	0.0378	4.34	0.11	0.0001	3.74	0.18	0.1540	3.81	0.16	0.1028
Sodium	4.98	0.34	6.78	0.23	0.0002	4.69	0.09	0.4418	4.98	0.35	1.0000	3.97	0.34	0.0330	5.00	0.14	0.9503	4.69	0.20	0.4555	4.93	0.18	0.8815
Refined Grains	6.78	0.30	5.72	0.23	0.0054	5.40	0.12	0.0001	7.02	0.33	0.5979	6.55	0.26	0.5711	4.95	0.08	<0.0001	4.63	0.21	<0.0001	4.20	0.16	<0.0001
SoFAAS Calories	9.42	0.58	6.46	0.41	0.0003	10.50	0.21	0.0992	10.80	0.63	0.1216	12.33	0.54	0.0004	10.65	0.16	0.0250	10.19	0.36	0.2374	10.00	0.34	0.3755
HEI-2010 Total Score	42.72	0.85	40.90	0.92	0.1926	46.06	0.48	0.0003	48.48	1.19	0.0005	50.58	1.02	<0.0001	46.02	0.44	0.0009	43.49	0.60	0.4102	44.33	0.65	0.1534

NHANES 2005–2010, *N* = 8,367

*LSM*: least square mean; *SE*: standard error; *P* = *p*: value of difference as compared to cluster 0 (no grains)

*SoFAAS*: solid fat, alcohol, added sugars

Covariates include age, gender, ethnicity, poverty income ratio, physical activity, current smoking status, and alcohol

When examining the subcomponents of HEI-2010 (Table 3), children and adolescents in the ‘crackers, salty snacks’, ‘pancakes, waffles, French toast and other grains’ and ‘quick breads’ had significantly lower total vegetable scores than subjects in the no grains pattern. Children and adolescents in all grain clusters examined had significantly greater scores for whole grains as compared to those not consuming grain foods, which indicated higher consumption of whole grains (see Table 3). Children and adolescents in the ‘cakes, cookies and pies’ grain cluster were the only cluster to show significantly higher scores for sodium intake relative to individuals not consuming grains. The lower HEI-2010 sub-component scores were more than offset with increased scores for those in the ‘yeast breads and rolls’, ‘cereal’, ‘pasta, cooked cereals and rice’ and ‘crackers and salty snacks’ clusters for total fruit, whole fruit and whole grains as compared to those not consuming grain foods. Additionally, children and adolescents consuming ‘yeast breads and rolls’, ‘pasta, cooked cereals, and rice’, ‘crackers and salty snacks’ and ‘quick breads’ had significantly higher scores for greens and beans, while the ‘cereals’ cluster showed higher dairy scores in comparison to children and adolescents in the no grains pattern. The significantly greater score for empty calories in the ‘pasta, cooked cereals, and rice’ cluster translates as fewer calories from solid fats, alcohol and added sugars), while those consuming ‘cakes, cookies and pies’ ingested more calories from solid fats, alcohol and added sugars relative to the no grains dietary pattern.

## Discussion

This is the first study that has identified various grain food patterns in US children and adolescents with reported associations between grain pattern consumption, energy and nutrient intakes and diet quality. The current data support that a variety of grain food patterns, including those recommended by dietary guidance and those that focus on enriched and fortified grain staples, are associated with greater nutrient intakes, including higher consumption of shortfall nutrients and nutrients of public health concern as identified by the 2015–2020 DGA [1], in comparison to an alternative dietary pattern that does not emphasize grain-based foods in children and adolescents. The findings from the present study are aligned with recently published data in American adults, where consumption of specific grain foods were associated with greater nutrient intakes, including greater consumption of shortfall nutrients and nutrients of public health concern. Several, but not all grain food patterns, were associated with improved diet quality compared to adults not consuming main grain groups. Adults consuming pasta, cooked cereals and rice also had lower body weights and smaller waist circumferences when compared to individuals not consuming grain foods [7].

**Several nutrients contributed by grain foods naturally or via fortification/enrichment, including folate, calcium, magnesium, fiber and iron are under consumed relative to IOM nutrition standards** **[** 17 **]**. **Dietary patterns that encourage nutrient-dense grain foods, with the concept of limiting sodium, total fat and sugar, may help shift population consumption in children and adolescents toward recommended intake levels for several shortfall nutrients identified by 2015 DGAC** **[** 17 **]**. **Additionally, creating positive habits including** **nutrient-dense** **dietary patterns that include whole and enriched grain consumption in earlier years may benefit health outcomes into adulthood** **[** 18, 19 **]**. Indeed, the current research in children and adolescents provides a sound rationale to support more specific dietary guidance for American children and adolescents about grain consumption rather than simply having two broad categories of recommended intakes that revolve around refined/enriched and whole grains. The current data illustrates how various enriched grain products contribute to daily nutrient intakes and overall diet quality. For example, we observed that children and adolescents consuming ‘yeast breads and rolls’, ‘cereals’, ‘pasta, cooked cereals and rice’, and ‘crackers and salty snacks’ grain patterns had a significantly higher diet quality, as measured by USDA’s HEI-2010 and dietary fiber intake was significantly greater in five of the eight patterns, ranging from 1.8 ‒ 2.8 g more daily fiber, as compared to those consuming no grain foods. It is rationale to suggest that these daily increases in dietary fiber can have a meaningful impact on public health initiatives by helping to minimize gaps in fiber consumption in children and adolescents. In fact, a recent study evaluating ten-year trends in fiber intakes using NHANES data from 2001–2010 in children and adolescents reported mean fiber intake to be 13.2 ± 0.1 g/day [20]. Thus, dietary fiber intake levels in children and adolescents continue to fall short of meeting dietary guidance based on recommendations set forth by the Institute of Medicine where fiber Adequate Intake in children 1 ‒ 8 years and children and adolescents 9–18 years is set at 19 ‒ 25 g/day and 26 ‒ 38 g/day, respectively [21].

Collaborative efforts from the American Heart Association, American College of Cardiology and The Obesity Society state that nearly one-third of children and youth are overweight or obese, further exacerbating poor health profiles and increasing risks for chronic diseases and their co-morbidities [22, 23]. In the current analyses, total fat intake was lower in, ‘cereals’ and ‘pasta, cooked cereals and rice’, and daily saturated fat intake was lower in many of the grain patterns examined, in comparison to the no grains food pattern. The range of saturated fat lowering per day translates to meaningful reductions when considering the U.S. Food and Drug Administration’s Daily Value (DV) for saturated fat; the mean lowering of saturated fat ranged from 1.5 ‒ 4.8 g per day which represents 7.5 ‒ 24% of the DV for adults and children ≥4 years of age consuming 2000 kcal/day. Taken collectively, some grain food patterns, comprised of both whole and enriched grains, can be beneficial in children and adolescents when considering dietary guidance and health outcomes.

Our results are aligned with previous observational findings that considered sources of nutrients in the US diet. When identifying the top food sources of nutrients, including both intrinsic and added to foods via fortification, results showed that grain foods represented the top five ranking food sources for folate, such that ready-to-eat cereals, yeast breads and rolls, pizza, pasta and crackers, popcorn, pretzels, and chips contributed 56.7 and 54.4% of folate to the diet of children and adolescents, respectively. Results were similar another shortfall nutrient, such that grain foods represented the top five food sources for iron in the diet of US children and adolescents, with ready-to eat cereals, yeast breads, pizza, cakes, cookies, and pies, and crackers, popcorn, pretzels, and chips cumulatively contributing 52.1 and 48.7% of iron [24].

The 2015–2020 DGA and 2015 Dietary Guidelines Advisory Committee (2015 DGAC) report further states ‘of the shortfall nutrients, calcium, vitamin D, fiber, and potassium also are classified as nutrients of public health concern because their under consumption has been linked in the scientific literature to adverse health outcomes” [21], a principle carried forward from the DGA 2010 policy document [25]. The 2015 DGAC [17] also reports that “if whole grains were consumed in the amounts recommended in the recommended food patterns, whole grains would provide substantial percentages of several key nutrients, such as about 32% of dietary fiber, 42% of iron, 35% of folate, 29% of magnesium and 16% of vitamin A”. While these nutrients levels represent significant contributions from whole grains, whole grain consumption alone can still leave a gap between consumption and recommendation levels. The 2005 DGAC reported that refined grains contribute substantial levels of key nutrients to food patterns, naming folate, iron, calcium, dietary fiber, thiamin, riboflavin and niacin [26], thus demonstrating the importance of consuming both enriched and whole grains. The committee further acknowledged that including only three ounce equivalents of whole grains daily with no refined grains in recommended food patterns would lower intake of many of key nutrients and potentially place specific populations at risk for nutrient inadequacy [26]; an argument which led the 2015 DGAC to conclude that consumption of whole grains with no substitutions would result in nutrient shortfalls [17]. The current analysis provides data linking different grain food patterns with nutrient intakes and concurrently we observe the adverse nutrient- and health-related outcomes when grain foods as a whole are eliminated from the diet. In many of the grain patterns examined, we see a better overall nutrient intake profile, which demonstrates the important dietary contributions made by different grain foods and emphasizes the importance of consuming a balance of whole grains, enriched and fortified grain products. Indeed, while some of the grain food clusters contributed nutrients to limit in the diet as identified by the 2015–2020 DGA [1], including saturated fat, added sugars, and sodium, several of the grain food patterns were associated with lower intakes of these nutrients and improved shortfall nutrients and diet quality. Such findings provide a rationale for more specific, evidence-based dietary guidance around grain consumption.

There are several limitations to the present analysis that deserve recognition. Data for energy and nutrient intakes, including values reported for diet quality, were obtained using 24-hour dietary recalls, which rely on study participant memory. While validated procedures are used to collect the data, recalled information may be inaccurate and biased from misreporting or memory challenges [27]. In addition, the current evidence, being observational, cannot establish a causal link between the different grain foods patterns examined and improvements in nutrient intakes and diet quality. However, a large strength of the current work stems from the use of NHANES, which is a large continuous survey that examines a nationally representative sample of about 5,000 individuals yearly by highly-trained medical personnel. Additionally, numerous covariates were used to adjust the data in an attempt to remove potential confounding scenarios. However, residual confounding may still exist and may explain some of the results reported. Lastly, we identified a small percentage of the population with no consumption of the main grain groups investigated, which served as the comparison group. While the comparison group was relevant for research purposes, it only represented 4% of the population, suggesting that further research is required.

## Conclusions

Several grain food dietary patterns in U.S. children and adolescents are associated with greater nutrient intakes, including greater consumption of shortfall nutrients and nutrients of public health concern as identified by the 2015–2020 DGA. Improved diet quality, as measured by USDA’s HEI-2010 was also linked to consumption of specific grain food patterns, including ‘pasta, cooked cereals and rice, ‘cereals’, ‘yeast breads and rolls’ and ‘crackers and salty snacks’, when compared to those children and adolescents not consuming grain food dietary pattern. Improved diet quality was due not only to the contribution of nutrients inherent in the grain, but also to those added through enrichment and fortification practices and those provided by natural food pairings such as cereal and dairy foods (i.e., milk). Overall, while some grain food patterns were associated with elevated sodium and added sugar, the present data also support that several grain food patterns can serve as part of a healthy dietary food pattern in children and adolescents, that accounts for 2015–2020 DGA dietary recommendations to reduce total fat, saturated fat and added sugar consumption, while concurrently increasing intake of shortfall nutrients and/or nutrients of concern, including iron, magnesium, dietary fiber, vitamin D, potassium and folate.
